# A146 UNDERWATER EMR WITHOUT SUBMUCOSAL INJECTION FOR COLONIC ANGIODYSPLASIA

**DOI:** 10.1093/jcag/gwad061.146

**Published:** 2024-02-14

**Authors:** R Yu, R Barclay

**Affiliations:** Internal Medicine, The University of British Columbia Faculty of Medicine, Vancouver, BC, Canada; Internal Medicine, The University of British Columbia Faculty of Medicine, Vancouver, BC, Canada

## Abstract

**Background:**

Angiodysplasia is a common source of overt or occult colonic bleeding. Lesions are amenable to endoscopic thermal treatment such as APC, which requires a dedicated device and carries a small risk of perforation. Underwater EMR without submucosal injection (UEMR) is safe and effective for colorectal polyps, and requires no special equipment.

**Aims:**

This study explores the use of UEMR to treat colonic angiodysplasia. We endeavoured to assess the effectiveness of treatment with UEMR in patients with this unique condition and further quantify the safety and adequacy of the obtained sample to achieve diagnosis. Further analyses were undertaken to determine the cost-burden of this method compared to comparable methods of treatment.

**Methods:**

Retrospective review of clinical database for patients who underwent UEMR for colonic angiodysplasia between 2021 and 2023.

Indications for treatment were iron deficiency anemia (N=4), Hematochezia (N=2), and melena (N=1).

Lesions were excised *en bloc* using standard UEMR technique without submucosal injection and soft-coagulation electrosurgical settings.

**Results:**

Six patients (5 females, mean age 76 ± 10) underwent colonoscopy with UEMR for treatment of angiodysplasia. One patient with extensive lesions required two endoscopic sessions; the others were treated during a single procedure. The median number of lesions was two; range, 1 to 13). A total of 24 lesions (size 7 ± 2 mm) were treated with UEMR. All lesions were located in the proximal colon (cecum, 14; ascending colon, 10). Histology of excised specimens confirmed a diagnosis of angiodysplasia.

There were no procedural complications. Overt bleeding and anemia resolved in all patients.

**Conclusions:**

In this series of patients, UEMR without submucosal injection was simple, safe and effective for treatment of proximal colon angiodysplasia. Advantages of this approach over conventional therapy with APC include reduced equipment needs, lower cost and histologic diagnosis. Further study comparing UEMR to conventional thermal modalities is warranted.

**Funding Agencies:**

None

